# Enhancing Catalytic Performance with Ni Foam-Coated Porous Ni Particles via 1-Butene Hydrogenation

**DOI:** 10.3390/ma18010195

**Published:** 2025-01-05

**Authors:** Dahee Park, Jung-Yeul Yun, Hye Young Koo, Yuchan Kim

**Affiliations:** Nano Materials Research Division, Korea Institute of Materials Science (KIMS), Changwon 51508, Republic of Korea

**Keywords:** porous Ni, Ni foam, 1-butene hydrogenation, interfacial active sites

## Abstract

The efficient hydrogenation of 1-butene is an industrially significant reaction for producing fuels and value-added chemicals. However, achieving high catalytic efficiency and stability remains challenging, particularly for cost-effective materials, such as Ni. In this study, we developed a porous Ni-coated Ni foam catalyst by electrostatic spray deposition to address these challenges. The catalyst exhibited a turnover frequency approximately 10 times higher than that of either porous Ni or Ni foam alone. This enhancement was attributed to the formation of interfacial active sites, which facilitated improved reactant adsorption and activation during hydrogenation. The electrostatic spray deposition technique ensured a uniform and controlled coating, enabling precise engineering of the catalyst structure and interface. The post-deposition heat treatment was further optimized to enhance structural integrity and catalytic performance. This study highlights the importance of interface engineering and structural optimization in catalyst design and provides valuable insights into the development of efficient Ni-based catalysts for industrial hydrogenation applications. These findings emphasize the potential of electrostatic spray deposition as a versatile method for fabricating advanced catalytic systems.

## 1. Introduction

The hydrogenation of olefins, such as 1-butene, is a crucial process in the production of fuels, polymers, and various high-value chemicals, making it an essential process in the chemical industry. Despite its importance, the development of catalysts having high efficiency, stability, and cost-effectiveness remains challenging. Ni-based catalysts are widely used for hydrogenation because of their robustness, affordability, and excellent catalytic activity [1,2,3]. Porous Ni has garnered significant attention as a catalyst because of its high density of active sites, which facilitates hydrogenation reactions [4,5,6]. However, its structural instability and performance limitations under prolonged reaction conditions hinder its widespread application.

An effective strategy to overcome these challenges involves combining porous nickel with nickel foam. As a supporting material, nickel foam provides notable advantages, including superior mechanical strength, outstanding electrical conductivity, and an extensive surface area. This combination results in unique interfacial active sites, which enhance the catalytic performance by improving reactant adsorption and activation while accelerating reaction kinetics [7,8,9]. These structural and interfacial synergies are particularly effective for hydrogenation reactions, where surface interactions critically influence the catalytic activity.

The fabrication of these composite structures requires advanced techniques to ensure their uniformity and stability. Electrostatic spray coating has emerged as an efficient method for the controlled deposition of porous Ni on Ni foam. This technique ensures homogeneous coating and precise control over the interface, which is crucial for maximizing the active-site exposure and catalytic performance [10,11,12]. Furthermore, heat treatment after coating can optimize the particle–Ni foam interfacial characteristics, enhancing the activity and durability of the catalyst. Recent studies have shown that interface tuning is crucial to enhancing the efficiency of Ni-based catalysts in industrial hydrogenation.

The hydrogenation of 1-butene, a critical process in the chemical industry, plays a vital role in producing high-purity alkanes used in fuels, polymers, and specialty chemicals. Thus, ensuring efficient and selective hydrogenation is essential for optimizing downstream production processes [13,14,15,16]. Porous nickel catalysts, characterized by abundant active sites and robust catalytic properties, demonstrate remarkable activity for 1-butene hydrogenation [6,15,17]. However, prolonged operation often leads to various issues, including structural degradation and performance loss, which can be mitigated by improving catalyst designs. In the present study, we addressed these challenges by combining porous nickel with nickel foam to create a composite catalyst having enhanced stability and reactivity. We leveraged the synergistic effects between the porous nickel layer and nickel foam support to devise a durable and efficient solution for 1-butene hydrogenation. The proposed approach has the potential for industrial applications owing to its cost-effectiveness and ability to improve catalyst performance [18,19,20,21,22].

In this study, powder-coated Ni foam structures were fabricated by electrostatic spray deposition. The process parameters, including the coating and post-treatment conditions, were systematically optimized to control the interfacial properties between the Ni particles and Ni foam. This study highlights the significance of interfacial engineering and process control in the development of advanced catalysts, providing new insights into the design of highly efficient and durable materials for hydrogenation reactions.

## 2. Materials and Methods

### 2.1. Synthesis of Porous Ni Particles

The porous Ni particles used in this study were synthesized following the method reported by Park et al. [6]. The synthesis involved spray pyrolysis of a precursor solution containing 0.1 M nickel acetate tetrahydrate (Ni(OCOCH_3_)_2_·4H_2_O) and 100 nm-sized polystyrene (PS) beads as a templating agent. The precursor solution was prepared by dissolving nickel acetate tetrahydrate in distilled water and adding PS beads, in a molar ratio of Ni:PS = 1:20. The mixture was stirred for 1 h at 300 rpm using a magnetic stirrer to ensure homogeneity. The prepared solution was aerosolized in a high-temperature furnace using a spray pyrolysis system. Nitrogen gas (purity ≥99.99%) was used as the carrier gas at a flow rate of 20 L/min to transport the aerosolized droplets into the furnace. The furnace temperature was maintained at 700 °C, with a controlled heating ramp of 10 °C/min to facilitate complete pyrolysis of the precursor components. The pyrolyzed particles were collected on a deposition plate at the furnace outlet to minimize contamination and agglomeration. To further enhance uniformity, process parameters such as solution feed rate and gas flow rate were carefully adjusted according to the precursor composition and operating conditions.

### 2.2. Deposition of Porous Ni Particles on Ni Foam

Porous NiO particles prepared by spray pyrolysis were deposited onto Ni foam by electrospray deposition (ESD). To prepare a uniformly dispersed solution, 1 wt% of the porous particles was added to ethanol (95%, Sigma-Aldrich, St. Louis, MO, USA) and stirred at 300 rpm for 1 h to prevent sedimentation and ensure consistent dispersion. The solution was delivered using a syringe pump to maintain a precise flow rate. The Ni foam substrate (10 × 10 × 0.3 cm^3^) was placed on aluminum foil under the nozzle, maintaining an optimal nozzle-to-foam distance of 3 cm. An electric field of 13 kV was applied via controlled droplet deposition onto the foam surface at a steady flow rate of 5 μL/min. The deposition process was performed in a sealed acrylic chamber under ambient conditions, and the humidity levels were carefully monitored to minimize solvent evaporation during spraying.

After the deposition, the samples were air-dried for 15 min and then subjected to heat treatment inside a box furnace at 600 °C, under a high-purity hydrogen atmosphere (≥99.999%), with a controlled heating rate of 5 °C/min). Via this heat treatment, the nickel oxide was reduced to metallic nickel, resulting in strong particle-to-foam bonding and removal of residual solvents. The Ni foam was subjected to ultrasonic cleaning in ethanol and deionized water via drying in a convection oven at 100 °C for 1 h to further improve its adhesiveness and uniformity. Additionally, the surface of the Ni foam was treated with low-pressure plasma for 5 min to enhance its wettability and allow an even distribution of particles. These process refinements resulted in a uniform coating of porous Ni particles on the Ni foam substrate, minimized particle aggregation, and improved the catalytic interface characteristics. The resulting system was suitable for advanced catalytic applications.

### 2.3. Characterization

The morphological and pore characteristics of the synthesized samples were examined by field-emission scanning electron microscopy (FE-SEM, JSM-7001F, JEOL Ltd., Tokyo, Japan) and FE-transmission electron microscopy (JEM-2100F (HR), JEOL Ltd., Tokyo, Japan). The crystal structure and composition of the samples were analyzed by X-ray diffraction (XRD, D/Max-2500VL, Rigaku Corporation, Tokyo, Japan) with Cu-Kα radiation (λ = 1.5418 Å). The oxidation states of the surface elements in the samples were analyzed by X-ray photoelectron spectroscopy (XPS) (Thermo Fisher Scientific K-Alpha and NEXSA instruments, Thermo Fisher Scientific Inc., Waltham, MA, USA). An Al Kα source was used for measurements, which utilized the adventitious C 1s peak at 284.6 eV.

### 2.4. 1-Butene Hydrogenation Reaction

The hydrogenation of 1-butene to butane was conducted in a flow reactor constructed from quartz tubing (inner diameter: 1.0 cm, outer diameter: 1.5 cm, and length: 50 cm) under atmospheric pressure. Figure S1 depicts the reactor and reaction scheme. A 50 mg sample was positioned at the center of the reactor using a quartz holder. A gas mixture comprising 1-butene and hydrogen was introduced into the reactor, with 1-butene delivered at a rate of 5 mL/min via a syringe pump preheated to 100 °C, while hydrogen was supplied at a flow rate of 100 mL/min. The reaction was performed at 100 °C under continuous hydrogen flow. The primary reaction product—butane—was quantified using gas chromatography.

## 3. Results and Discussion

### 3.1. Fabrication of Porous Ni-Coated Ni Foam

**Figure 1:**
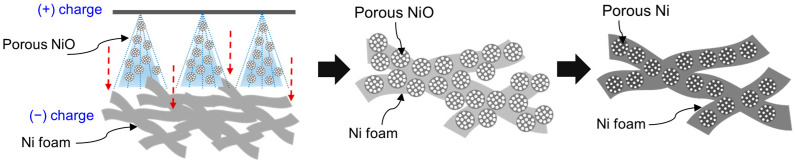
Scheme of preparation process of porous Ni-coated Ni foam via ESD and reduction.

Figure 1 illustrates the preparation of porous Ni-coated Ni foam using an ESD method followed by a reduction treatment. This approach was designed to deposit porous NiO particles onto Ni foam, which served as a stable catalyst with reduced material usage for catalytic reactions. In the initial step, the porous NiO particles were deposited on the Ni foam surface via ESD. A positively charged spray of NiO particles was directed toward the negatively charged Ni foam, which was driven by the electrostatic force difference between the powder solution and the foam substrate [23]. The ESD process ensured the uniform deposition of porous NiO particles by optimizing critical parameters, such as the applied voltage, solution concentration, number of nozzles, and solution injection rate [24]. These carefully controlled conditions ensured a consistent NiO coating across the foam surface, creating a uniform catalyst layer. Following deposition, the coated Ni foam underwent a high-temperature reduction process in a hydrogen atmosphere. During this step, the NiO particles were converted into porous metallic Ni as oxygen was removed from the NiO structure. This transformation enhanced the electrical conductivity and catalytic activity of the material while maintaining its porous structure. The reduction process also facilitated strong adhesion between the porous Ni and the foam substrate, minimizing particle detachment during the operation [25]. The final product, a porous Ni-coated Ni foam, exhibited a large surface area and a well-integrated porous Ni layer, offering excellent catalytic properties and structural stability. The ESD method enabled precise control over the catalyst deposition by reducing the amount of material required while ensuring uniform particle distribution and effective utilization of the active sites [26]. These features render the porous Ni-coated Ni foam highly effective for catalytic applications, such as hydrogenation, where durability and efficiency are critical.

**Figure 2:**
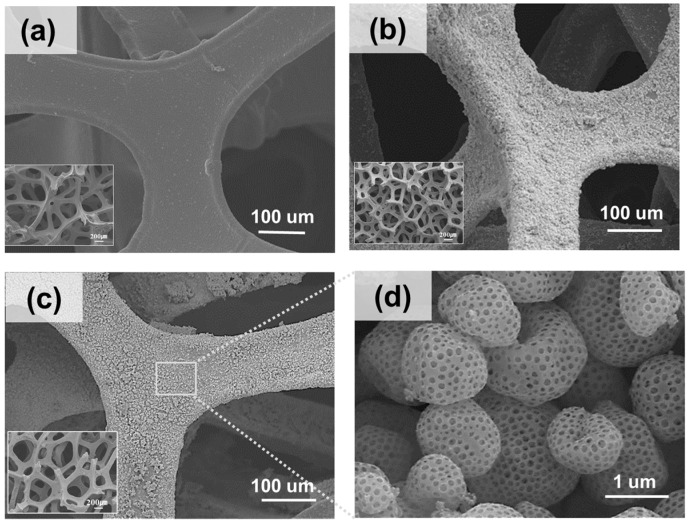
SEM images of (**a**) Ni foam (ref), (**b**) porous NiO-deposited Ni foam, and (**c**) porous Ni-deposited Ni foam. (**d**) Enlarged image of porous Ni particles after reduction process.

Figure 2a shows the SEM image of the pristine Ni foam, which exhibits a smooth and clean surface without any deposited particles. The interconnected framework of the Ni foam provides a robust mechanical structure and large surface area, making it a suitable support for catalyst deposition [27]. The Ni foam surface becomes rough after the ESD of porous NiO owing to the uniform coverage of the porous NiO particles, which are evenly distributed across the foam (Figure 2b). The inset presents the fine structure of the porous NiO particles, which exhibit a large surface area and high porosity, which are the key characteristics responsible for enhancing catalytic performance [28]. A uniform distribution of particles is achieved by optimizing the electrospray conditions, which ensure effective utilization of the support structure. Figure 2c shows the Ni foam after reduction, where the porous NiO particles are converted into porous metallic Ni. The foam retained the rough surface, as shown in Figure 2b, with a well-distributed layer of the porous metallic Ni. The inset provides a closer view, confirming the preservation of the porous structure during reduction. This porous metallic Ni layer enhanced the number of active sites available for catalytic reactions, improving the overall performance of the material [29]. Figure 2d shows an enlarged image of the porous Ni particles after reduction. The porous particles exhibited well-defined architectures with interconnected pores, facilitating the diffusion of reactants and products during catalytic reactions. This highly porous structure improved the accessibility of active sites and ensured strong adhesion to the Ni foam substrate, contributing to the mechanical stability of the material during operation [30]. These SEM images collectively demonstrate that ESD, combined with a hydrogen reduction step, successfully transformed the pristine Ni foam into a robust and porous Ni-coated material. Therefore, the resulting structure, which is characterized by high porosity, uniform distribution, and strong adhesion, is ideal for applications requiring efficient and stable catalysts, such as hydrogenation reactions and other catalytic processes.

### 3.2. Influence of the Reduction Temperature and Coating Conditions on the Porous Ni Particles

**Figure 3:**
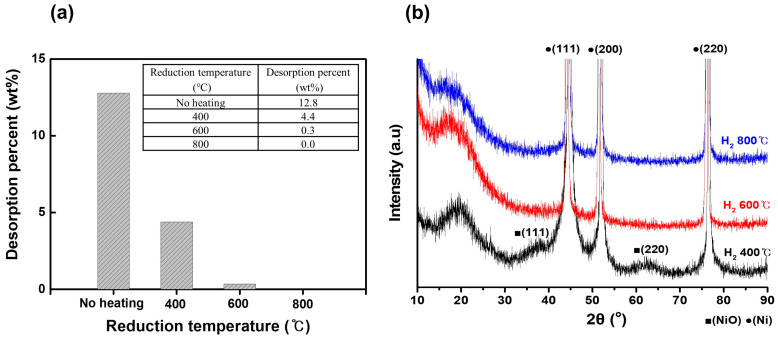
(**a**) Desorption weight percentage and (**b**) XRD pattern of porous Ni particles on Ni foam depending on reduction temperature at hydrogen atmosphere.

Figure 3 illustrates the effect of the coating conditions and reduction temperature on the properties of the porous Ni particles supported on the Ni foam. The amount of coating and the reduction process were controlled by varying the concentrations of the solution used for coating and the reduction temperature. The results are discussed in terms of the desorption weight percentage (a) and XRD patterns (b). Figure 3a shows the desorption weight percentage data, revealing the influence of the reduction temperature on the adhesion of the Ni particles to the foam surface after sonication. Without heating, the desorption percentage reached its highest value at 12.8 wt%, indicating poor adhesion and a substantial loss of particles upon sonication [31]. At 400 °C, the desorption percentage decreased to 4.4 wt%. At 600 °C, the desorption percentage decreased to nearly zero (0.3 wt%), indicating strong particle adhesion with negligible detachment after sonication. This finding suggests that reduction at 600 °C effectively anchored the particles onto the Ni foam. No desorption was observed at 800 °C, indicating the complete stabilization of particles and highlighting the critical role of the reduction temperature in enhancing the adhesion and integration of particles on the Ni foam surface. Figure 3b shows the XRD patterns, which provide insights into the structural and phase transitions of the Ni particles depending on the reduction temperature. At 400 °C, the peaks corresponding to NiO at approximately 37°, 43°, and 62° indicate that the particles were primarily composed of NiO [32]. At 600 °C, the intensity of these NiO peaks decreased significantly, and peaks corresponding to metallic Ni appeared at 44.5°, 51.8°, and 76.4°, signifying a phase transition from NiO to metallic Ni [32]. Notably, no particle aggregation was observed at this temperature, suggesting that reduction facilitated a uniform phase transformation without particle coalescence. At 800 °C, the NiO peaks disappeared completely, with a predominance of the metallic Ni peaks, confirming the complete reduction of NiO to Ni and the preservation of particle structure. These results indicate that at 600 °C particle desorption was nearly eliminated, and a clear phase transition from NiO to metallic Ni occurred, without significant particle aggregation. This finding demonstrates that 600 °C was an optimal reduction temperature for achieving strong particle adhesion and effective phase transformation while maintaining the structural integrity of particles. The ability to control the coating amount and reduction process by adjusting the solution concentration and reduction temperature underscores the importance of these parameters in tailoring the properties of the Ni-coated Ni foam for catalytic or other applications.

**Figure 4:**
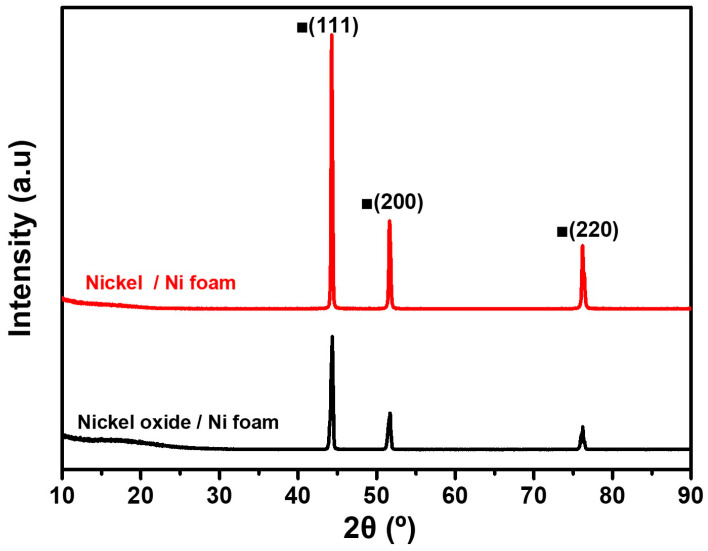
XRD pattern for porous Ni oxide deposited on Ni foam (black) and porous Ni deposited on Ni foam (red) after reduction.

Figure 4 shows the XRD patterns of the porous Ni oxide (black) and porous Ni (red) deposited on the Ni foam after reduction, following the optimized conditions identified in Figure 3. These patterns highlight the transformation of the NiO into the metallic Ni and illustrate the effectiveness of reduction. The XRD pattern of the porous Ni oxide exhibits distinct peaks at approximately 37°, 43°, and 62°, corresponding to the (111), (200), and (220) crystal planes of NiO, respectively [32]. These peaks confirm the crystalline nature of Ni oxide before reduction, consistent with the previously reported NiO structures [32]. After reduction, the XRD pattern of the porous Ni exhibits sharp peaks at 44.5°, 51.8°, and 76.4°, corresponding to the (111), (200), and (220) crystal planes of metallic Ni [32]. The disappearance of the NiO peaks and the emergence of only metallic Ni peaks confirms the successful conversion of Ni oxide to metallic Ni under optimized reduction conditions. This complete phase transition corroborates the results from Figure 3, where reduction at 600 °C eliminates the adsorbed species and enables full conversion. The high intensity and sharpness of the metallic Ni peaks indicates a well-formed crystalline structure, suggesting that the reduction process enhanced the crystallinity of the Ni particles while ensuring strong adhesion to the Ni foam substrate. In addition, the absence of secondary phases, such as mixed oxides or intermediate species, reflects the efficiency of the reduction process in achieving a clean and uniform transformation without aggregation or structural defects. This result demonstrates that the optimized reduction process effectively converted NiO to metallic Ni while preserving the structural integrity of the particles. This precise phase control and crystallinity enhancement rendered the reduced porous Ni material highly suitable for catalytic and other advanced applications. These findings emphasize the importance of carefully controlling the reduction conditions for producing high-quality Ni coatings on the Ni foam.

### 3.3. Oxidation States of the Porous Ni and Ni Oxide-Coated Ni Foam

**Figure 5:**
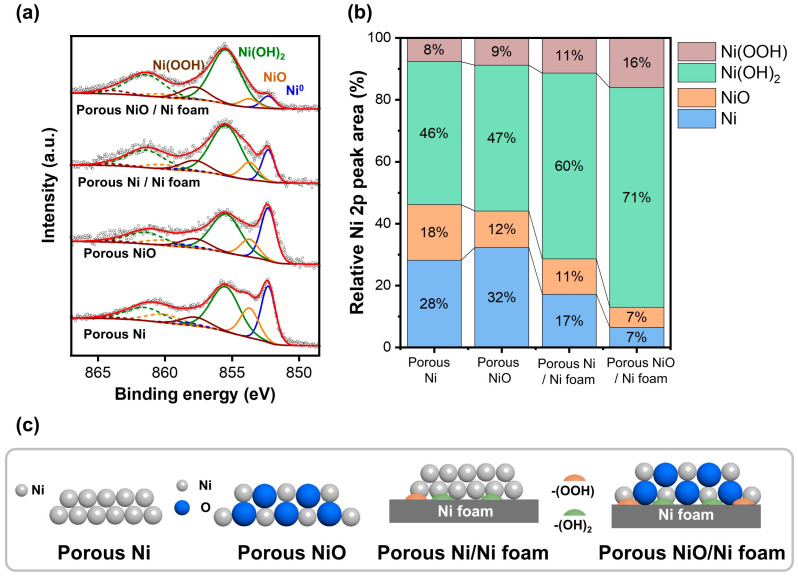
XPS profiles of porous NiO, Ni, NiO-coated Ni foam, and Ni-coated Ni foam. (**a**) XPS intensity, (**b**) Ni 2p peak area (%), and (**c**) scheme of oxidation of samples.

Figure 5 shows the XPS results of the porous Ni and Ni oxide (NiO) samples, highlighting their surface chemical states and catalytic properties. Figure 5a shows the XPS Ni 2p profiles of the four samples, porous Ni, NiO, NiO-coated Ni foam, and Ni-coated Ni foam, revealing peaks corresponding to different oxidation states, including metallic Ni, NiO, Ni(OH)_2_, and Ni(OOH), respectively. The metallic Ni peak was observed at approximately 852.6 eV, NiO near 854.0 eV, Ni(OH)_2_ at approximately 855.6 eV, and Ni(OOH) at approximately 856.5 eV [33]. These variations in the peak positions and intensities provide critical insights into the surface oxidation states and chemical compositions of the samples, which are essential for understanding their catalytic behavior. Figure 5b shows the relative peak areas of Ni 2p (%), highlighting the differences in the oxidation state distributions of the samples. Ni^0^, NiO, Ni(OH)_2_, and Ni(OOH) account for 46%, 28%, 18%, and 8% of the porous Ni, respectively. For porous NiO, NiO was dominant at 47%, followed by Ni(OH)_2_ (32%), Ni^0^ (12%), and Ni(OOH) (9%). The porous Ni-coated Ni foam exhibited a higher proportion of Ni^0^ (60%) and NiO (17%), with smaller contributions from Ni(OH)_2_ (11%) and Ni(OOH) (11%). The porous NiO-coated Ni foam was characterized by 7% Ni^0^, 16% NiO, 71% Ni(OH)_2_, and 7% Ni(OOH). These differences emphasize the variation in the oxidation states and chemical compositions resulting from coating and reduction. The schematic in Figure 5c illustrates the oxidation processes and structural changes in the porous Ni and NiO samples. The superior catalytic performance of the porous Ni/Ni foam in the hydrogenation reactions can be attributed to its optimized surface chemistry and composition. The XPS data reveal a balanced coexistence of metallic Ni and NiO in this sample, which is essential for the catalytic activity. Metallic Ni facilitates the adsorption and activation of the hydrogen molecules, whereas NiO stabilizes the reaction intermediates and supports the reaction pathways. In addition, the moderate presence of Ni(OH)_2_ contributes to the catalytic stability and efficiency [3,34]. The high electrical conductivity of the metallic Ni and oxygen-supplying capability of the NiO create an ideal environment for hydrogenation reactions. Furthermore, the porous structure enhances the catalyst performance by increasing the surface area and active-site density and improving the contact between the reactants and the catalyst. These combined factors explain the superior catalytic properties of the porous Ni/Ni foam, making it an excellent material for hydrogenation applications.

### 3.4. Catalytic Performance of the Ni Foam Under 1-Butene Hydrogenation Reaction

**Figure 6:**
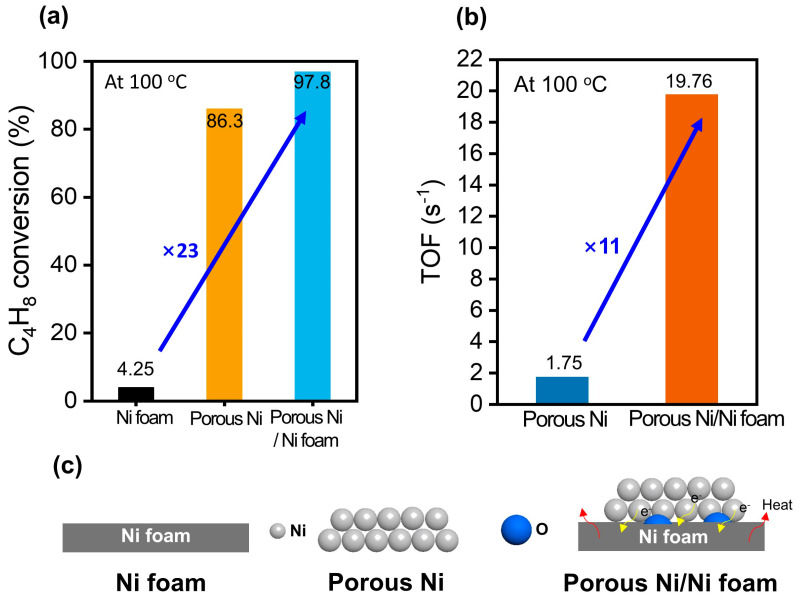
1-Butene hydrogenation reaction at 100 °C: (**a**) 1-butane conversion of Ni foam, porous Ni, and porous Ni-coated Ni foam; (**b**) TOF of porous Ni and Ni-coated Ni foam; (**c**) scheme of Ni foam, porous Ni, and porous Ni-coated Ni foam.

Figure 6 illustrates the catalytic performance of the Ni foam, porous Ni, and porous Ni-coated Ni foam during the hydrogenation of 1-butene at 100 °C. The analysis focuses on conversion efficiency, turnover frequency (TOF), and structural characteristics. The conversion efficiency of the Ni foam (4.25%) is significantly lower than those of the porous Ni (86.3%) and porous Ni-coated Ni foam (97.8%; Figure 6a). The 23-fold improvement in the conversion efficiency of the porous Ni-coated Ni foam highlights the crucial role of the porous structure and additional Ni coating in enhancing the catalytic performance. Previous studies have reported similar findings, indicating that porous structures increase the number of active sites available and strengthen reactant interactions, thereby boosting catalytic activity [35]. This enhancement is attributed to the increased surface area and numerous accessible active sites provided by the tailored material design. The porous Ni-coated Ni foam demonstrated superior catalytic activity and high stability under prolonged reaction conditions, demonstrating potential for industrial applications. Such stability improvements are consistent with the results obtained in previous studies, which show that metal coatings on porous substrates mitigate structural degradation during catalytic processes, thereby maintaining high performance over time [36]. Figure 6b highlights the TOF values that further confirm the superior performance of the porous Ni-coated Ni foam. The TOF is calculated as [37]:

(1) $$TOF=\frac{Product\;formation\;rate(\frac{mol}{s})}{moles\;of\;active\;Ni\;sites\;(mol)}$$

The porous Ni-coated Ni foam exhibited a TOF of 19.76 s^−1^, which was 11 times higher than that of porous Ni (1.75 s^−1^). This improvement underscores the efficient utilization of active Ni sites, facilitated by the synergy between the increased surface area and optimally dispersed Ni species. The Brunauer–Emmett–Teller (BET) surface area values for the Ni and porous Ni-coated Ni foams (Table S1) were utilized to calculate the moles of active Ni sites and derive the TOF values. The substantial increase in the BET surface areas of the Ni-coated Ni and Ni foams (0.245 and 0.133 m^2^/g) was attributed to the porous structure and coating. These results reveal a robust correlation between the material’s structural properties and its catalytic performance, aligning with those of other studies, which reported enhanced TOFs in catalytic applications realized via porous structure incorporation and tailored Ni distributions [38].

The additional Ni coating facilitates hydrogen adsorption and activation, which are crucial steps in hydrogenation reactions. Figure 6c illustrates the structural differences among the three catalysts. Ni foam, with its smooth, nonporous surface, provides a limited number of active sites, thus exhibiting poor catalytic performance. Porous Ni offers a relatively larger surface area and more accessible active sites, delivering moderate performance. The porous Ni-coated Ni foam combines these advantages, incorporating an additional Ni layer to increase the density and number of accessible active sites. This approach has been validated in various studies, which have demonstrated that combining porous structures with metal coatings can substantially improve catalytic efficiency and reduce reaction barriers [39]. This combination enhances catalytic efficiency and promotes superior interaction with reactants, improving the hydrogenation performance of catalysts. Table S2 summarizes the applications of metal foams as catalysts, demonstrating the synergistic effects of metal foams as support structures in catalytic reactions as well as their contribution to mechanical stability enhancement and reactant diffusion. Specifically, the increased surface area and improved thermal conductivity of metal foams are attributed to their high reaction rates and good selectivity, which further evidence the versatility of metal foams as catalyst supports. In conclusion, the porous Ni-coated Ni foam exhibited exceptional activity in the hydrogenation of 1-butene, outperforming both Ni foam and porous Ni. Its optimized structure, increased active-site density, and efficient Ni utilization resulted in high conversion rates and TOFs, which confirmed its effectiveness as a catalyst for hydrogenation reactions. These findings are consistent with results in the existing literature and highlight the potential of porous Ni-coated Ni foams as efficient catalysts for industrial applications, particularly in processes requiring high stability and efficiency under demanding conditions.

## 4. Conclusions

This study demonstrates the successful preparation and characterization of a porous Ni-coated Ni foam through a combination of ESD and hydrogen reduction. The ESD process enabled the uniform and precise coating of porous NiO particles onto the Ni foam substrate, driven by electrostatic interactions. The deposited particles exhibited a well-distributed porous structure, which was retained after reduction. The post-deposition reduction in a hydrogen atmosphere effectively converted the porous NiO into metallic Ni, with optimized conditions at 600 °C, ensuring complete phase transformation while preserving the porous morphology and maintaining strong adhesion to the foam. Structural analysis using SEM confirmed the transformation of the smooth Ni foam surface into a rough, porous, Ni-coated material with a large surface area and interconnected pores, which is ideal for its catalytic applications. The XRD results validated the phase transition from NiO to Ni during reduction, with no evidence of the intermediate phases or particle aggregation. XPS analysis further revealed a balanced presence of the metallic Ni and NiO in the reduced samples, highlighting the importance of surface composition in achieving superior catalytic performance. The porous Ni-coated Ni foam produced in this study demonstrated a combination of a large surface area, abundant active sites, and robust structural stability, making it highly effective for catalytic applications, such as hydrogenation. The ESD process, coupled with precise reduction conditions, provides a scalable and versatile method for tailoring material properties to meet specific application requirements. This approach established a strong foundation for the development of advanced catalytic materials with enhanced efficiency, stability, and durability.

## Data Availability

The datasets are available on reasonable request.

## Supplementary Materials

The following supporting information can be downloaded at https://www.mdpi.com/article/10.3390/ma18010195/s1. Figure S1: Schematic of the reactor and reaction procedure; Table S1: BET surface areas and density values; Table S2: Applications of metal foams as catalysts. References [40,41,42,43,44] are cited in the Supplementary Materials.

## Abbreviations

The following abbreviations are used in this manuscript:

PS	Polystyrene
SP	Spray pyrolysis
ESD	Electrospray deposition
SEM	Scanning electron microscopy
XRD	X-ray diffraction
BET	Brunauer–Emmett–Teller
XPS	X-ray photoelectron spectroscopy
