# Supplementary material for: Enhancing Catalytic Performance with Ni Foam-Coated Porous Ni Particles via 1-Butene Hydrogenation
Source: Materials (Basel). 2025 Jan 5;18(1):195. doi: 10.3390/ma18010195 (PMC11721857; doi:10.3390/ma18010195)
Supplement: Supplementary file 1 [file materials-18-00195-s001.zip › materials-3385306-supplementary.pdf]

Supplementary Materials

Enhancing Catalytic Performance With Ni Foam-Coated Porous Ni Particles via 1-Butene Hydrogenation

Dahee Park*, Jung-Yeul Yun, Hye Young Koo, Yuchan Kim

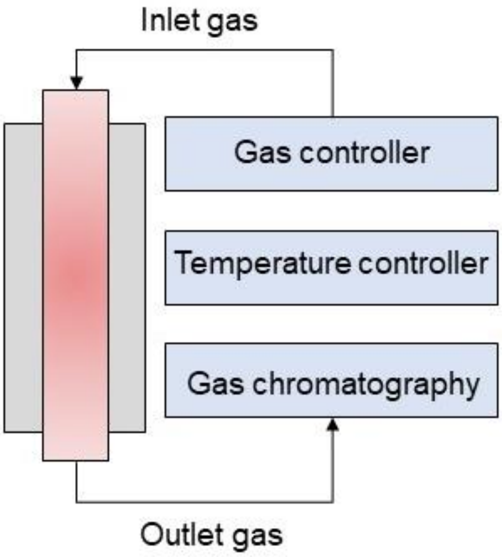

Figure S1. Schematic of the reactor and reaction procedure.

Table S1. BET surface areas and density values.

	BET (m^2/g)	Density (g/cm^3)	BET (m^2/L)
Ni foam	0.133	0.333	44.300
Porous Ni / Ni foam	0.245	0.361	88.583

Table S2. Applications of metal foams as catalysts.

Foam type	Reaction type	Performance	Reference
Copper foam (Cu foam)	CO_2 electroreduction	High selectivity for hydrocarbon production	[1]
Iron foam (Fe foam)	Fischer–Tropsch synthesis	Improved syngas conversion efficiency	[2]
Platinum-coated metal foam (Pt foam)	Ammonia oxidation	High catalytic activity and stability	[2]
Cobalt foam (Co foam)	Oxygen evolution reaction	Enhanced oxygen evolution efficiency	[3]
Aluminum foam (Al foam)	Hydrocarbon cracking	Superior cracking efficiency with thermal stability	[2]
Titanium foam (Ti foam)	Hydrogen storage	Increased hydrogen uptake capacity	[4]
Stainless-steel foam (SS foam)	Desulfurization of fuels	Effective sulfur removal under harsh conditions	[2]
Zinc foam (Zn foam)	Zn–air Batteries	High energy density and rechargeability	[2]
Palladium-coated foam (Pd foam)	Hydrogenation reactions	Selective hydrogenation with high conversion rates	[5]

References

[1] Cai, F.F.; Sarac, B.; Akman, A.; Londoño, J.J.; Gümrükcü, S.; Schweiger, L.; Hantusch, M.; Schroers, J.; Blatter, A.; Gebert, A.; Spieckermann, F. Nano-patterned Pt-based Metallic Glass Electrocatalysts With In-Situ Copper Oxide Foam for Enhanced Hydrogen Evolution. *arXiv preprint arXiv:2406.14079* **2024**.

[2] Walther, G.; Gaitzsch, U.; Büttner, T.; Kieback, B.; Weissgaerber, T.; Kolvenbach, R.; Lincke, M. Applications of Metal Foam as Catalyst Carrier. *Proc. Euro PM* **2017**.

[3] Pan, S.; Mao, X.; Yu, J.; Hao, L.; Du, A.; Li, B. Remarkably Improved Oxygen Evolution Reaction Activity of Cobalt Oxides by an Fe Ion Solution Immersion Process. *Inorg. Chem. Front.* **2020**, *7*(*18*), 3327–3339.

[4] Kibis, L.S.; Svintsitskiy, D.A.; Ovsyuk, I.Y.; Kardash, T.Y.; Romanenko, A.V.; Boronin, A.I. Modification of Physicochemical Properties of Platinum-Titanium Catalysts for Ammonia Slip Oxidation. *J. Struct. Chem.* **2024**, *65*(1), 125–137.

[5] Gilbert, D.A.; Burks, E.C.; Ushakov, S.V.; Abellan, P.; Arslan, I.; Felter, T.E.; Navrotsky, A.; Liu, K. Tunable Low Density Palladium Nanowire Foams. *Chem. Mater.* **2017**, *29*(*22*), 9814–9818.
